# Fabrication of Gelatin Methacrylate (GelMA) Scaffolds with Nano- and Micro-Topographical and Morphological Features

**DOI:** 10.3390/nano9010120

**Published:** 2019-01-18

**Authors:** Ana Agustina Aldana, Laura Malatto, Muhammad Atiq Ur Rehman, Aldo Roberto Boccaccini, Gustavo Abel Abraham

**Affiliations:** 1 Instituto de Investigaciones en Ciencia y Tecnología de Materiales, INTEMA (UNMdP-CONICET), Av. Juan B. Justo 4302, Mar del Plata B7608FDQ, Buenos Aires, Argentina; aaldana@fi.mdp.edu.ar (A.A.A.); gabraham@fi.mdp.edu.ar (G.A.A.); 2 Instituto Nacional de Tecnología Industrial, Centro de Micro y Nanoelectrónica del Bicentenario (INTI-CMNB), Av. Gral. Paz 5445, San Martin B1650KNA, Buenos Aires, Argentina; laura@inti.gob.ar; 3 Institute of Biomaterials, Department of Materials Science and Engineering, University of Erlangen-Nuremberg, 91058 Erlangen, Germany; muhammad.rehman.ur@fau.de; 4 Department of Materials Science and Engineering, Institute of Space Technology Islamabad, 1, Islamabad Highway, Islamabad 44000, Pakistan

**Keywords:** biomimetic scaffolds, gelatin, electrospinning, micromolding, biomaterials

## Abstract

The design of biomimetic biomaterials for cell culture has become a great tool to study and understand cell behavior, tissue degradation, and lesion. Topographical and morphological features play an important role in modulating cell behavior. In this study, a dual methodology was evaluated to generate novel gelatin methacrylate (GelMA)-based scaffolds with nano and micro topographical and morphological features. First, electrospinning parameters and crosslinking processes were optimized to obtain electrospun nanofibrous scaffolds. GelMA mats were characterized by SEM, FTIR, DSC, TGA, contact angle, and water uptake. Various nanofibrous GelMA mats with defect-free fibers and stability in aqueous media were obtained. Then, micropatterned molds produced by photolithography were used as collectors in the electrospinning process. Thus, biocompatible GelMA nanofibrous scaffolds with micro-patterns that mimic extracellular matrix were obtained successfully by combining two micro/nanofabrication techniques, electrospinning, and micromolding. Taking into account the cell viability results, the methodology used in this study could be considered a valuable tool to develop patterned GelMA based nanofibrous scaffolds for cell culture and tissue engineering.

## 1. Introduction

The design of biomimetic biomaterials as scaffolds for cell culture is a powerful tool for studying and understanding fundamental cell behavior, specific tissue environment, degradation, and reasons for tissue damage [1]. Scaffolding structures should mimic not only biological properties of extracellular matrix (ECM), but also morphological and topographical features [2,3,4]. The ECM directs and modulates cell behavior, is composed of fibrous proteins (mainly collagens and elastin), glycosaminoglycans (GAGs), proteoglycans, and glycoproteins [5,6].

In order to emulate the natural structure of the ECM, different technologies have been developed. Current techniques for generating topographical features on polymeric scaffolds for cell culture, especially those with nano-scale resolution, are typically complex and expensive. Thus, a simple and tunable fabrication method for the production of patterned biomimetic scaffold is still pending. By using electrospinning technology, it is possible to obtain micro- and/or nano-fibrous mats that mimic ECM [7,8]. The optimization of different processing parameters and the use of post-processing treatments allow handling dimensions, porosity, morphology, and the spatial arrangement of nanofibers. A huge variety of natural and/or synthetic polymeric solutions has been electrospun. Composition and processing techniques determine the scaffold architecture, mechanical performance, degradation rate, and cell-material interactions. Aligned and randomly oriented electrospun mats have been also developed to study how morphology affects cell behavior. Gao et al. studied the influence of aligned and randomly oriented fibrous gelatin/PLLA scaffolds to guide the growth of corneal stroma cells [9]. The aligned scaffold not only increased cell viability more significantly than that in a randomly oriented scaffold, but it also provided an external stimulus for the orderly arrangement of cells. Similar results were observed by Shalumon et al., who prepared aligned and randomly PLLA/gelatin nanofibrous scaffolds [10]. In these structures, an increase in viability and proliferation of human umbilical vein endothelial cells (HUVECs) and smooth muscle cells (SMCs) was observed.

On the other hand, substrates with various micro- and nano-features such as lines, wells, and holes among others have been explored to introduce significant effects on cell behavior [11]. Most of these reports relate the topographical features with cell orientation, migration, morphology, proliferation, cell gene expression, and differentiation [12,13,14].

The main goal of this work is to design gelatin-based scaffolds with micro and nano-topographical and morphological features, achieving a high resolution, and performance with low cost. In addition, gelatin, a biocompatible and biofunctional polymer, and benign solvents (Class 3 according to ICH guidelines) [15] are used for scaffold fabrication. Gelatin is an inexpensive biomacromolecule obtained from denatured collagen, and presents integrin cell-binding motifs, such as RGD and matrix metalloproteinases (MMP) degradable sites [16,17]. Compared to native collagen, gelatin has lower antigenicity and less batch-to-batch variation due to the denaturation process, in which tertiary protein structures are removed. Functionalization of amine-containing groups of gelatin with methacrylate groups was used to provide a photopolymerizable biomaterial named GelMA that has been widely investigated for cell-based studies and tissue engineering applications [18,19,20,21,22]. Crosslinking of the methacrylic side groups results in hydrogels with stiffness and density that can be controlled by varying the polymer dry mass, degree of functionalization, photo-initiator concentration, ultraviolet (UV) intensity, and exposure time. In this work, electrospinning and photolithography techniques were used to design 3D scaffolds with novel topographical features in micro- and nanoscale, while UV exposition time was varied. Moreover, the use of micropatterned molds with different sizes as collectors in the electrospinning process is proposed to produce electrospun fibrous mats.

## 2. Materials and Methods

### 2.1. Materials

Gelatin type A from porcine skin Gel Strength 300, methacrylic anhydride (MAA), and glacial acetic acid (AA) were purchased from Aldrich (Darmstadt, Germany). The photoinitiator 1-[4-(2-hydroxyethoxy)phenyl]-2-hydroxy-2-methyl-1-propan-1-one (Irgacure^®^2959) was kindly provided by BASF (Nienburg, Germany). Phosphate-buffered saline (PBS) was freshly prepared in the laboratory.

### 2.2. Synthesis of GelMA

The preparation of GelMA has been recently described by the authors [23]. Briefly, gelatin was dissolved in PBS (pH 7.4) at a concentration of 1% (wt/v) and 50 °C. Then, a predetermined amount of methacrylic anhydride was added, under vigorously magnetic stirring conditions. The mixture was left to react for one hour at 50 °C and was afterwards dialyzed by using a 12–14 kDa cutoff membrane against distilled water for several days. Finally, functionalized gelatin was frozen and freeze-dried. The degree of methacrylation, defined as the percent of amine groups converted to methacrylamide groups, was determined by Habeeb’s test [24]. Finally, GelMA was characterized by ^1^H-NMR (Bruker 400 MHz NMR spectrometer, Bruker Biospin, Rheinstetten, Germany) and FTIR spectroscopy (Mattson Instruments Inc., model Genesis II, Madison, WI, USA).

### 2.3. Fabrication of Electrospun GelMA Nanofibers

A predetermined amount of GelMA was completely dissolved in acetic acid at a 250 mg/mL concentration. Then, Irgacure 2959 was added to GelMA solution. The solution was electrospun through a blunt 18-gauge stainless steel needle onto an aluminum collector plate 10 cm away. A solution flow rate of 0.2 mL/h and an applied high-voltage of 12 kV were used. All experiments were carried out at room temperature and a relative humidity of 50%.

### 2.4. Photocrosslinking of GelMA Nanofibers

The obtained electrospun GelMA scaffolds were crosslinked by UV irradiation. Fibrous meshes were initially wetted in anhydrous ethanol and then exposed to UV light (UVL-28 lamp, 365 nm), during different time periods (0, 6, 9 and 12 min) at 2.5 cm (named NG-0UV, NG-6UV, NG-9UV and NG-12UV, respectively). After crosslinking treatment, samples were removed from aluminum foil and dried at room temperature in a vacuum oven.

### 2.5. Fabrication of Micropatterned Molds

Master mold layouts were designed using L-EditTM from Tanner EDA and printed on polyester-based film using a 3600 DPI printer. Molds were then created on silicon wafers by spin-coating SU8 2000 negative photoresist (10 s at 500 rpm and 30 s at 3000 rpm, Microchem Inc., Westborough, MA, USA). Wafers were previously cleaned with the standard piranha solution. The coated wafers were then soft-baked on a hot plate for 20 min at 65 °C, followed by 25 min at 95 °C, before exposing the photoresist through the photomask. Near UV (365 nm) was applied with an exposure dose of 650 mJ/cm^2^ (EVG 620, EV Group). Post exposure bake conditions were 5 min at 65 °C, followed by 10 min at 95 °C. Molds were finished by 10 min of immersion on the developer (MicroChem’s SU-8 Developer) with strong agitation, rinsed with isopropyl alcohol, and dried with nitrogen stream.

### 2.6. Fabrication of Micropatterned Nanofibrous GelMA Scaffolds

The electrospinning of micropatterned GelMA scaffolds was carried out by using the same procedure as described above except that the collector plate consisted in a micropatterned mold obtained by photolithography. After electrospinning, the meshes were crosslinked by UV irradiation for 9 min just as it was previously described.

### 2.7. Morphology Characterization

Surface morphology of the nanofibrous scaffolds before and after crosslinking was examined by scanning electron microscopy (SEM). Samples were placed on double-sided graphite tape, attached onto a metal surface, and sputter-coated with gold for 10 s. SEM micrographs were acquired with different magnifications using a SEM (Jeol USA Inc., model JSM-6460LV, Peabody, MA, USA). The average fiber diameter and fiber diameter distribution were estimated from SEM images, using ImagePro-Plus 6.0^®^ software.

### 2.8. Infrared Analysis

Chemical composition of the gelatin scaffolds was assessed by Fourier transform infrared spectroscopy with attenuated total reflectance (FTIR-ATR) using a Nicolet 6700 Thermo Scientific (Waltham, MA, USA) spectrometer equipped with a diamond crystal at a nominal incidence angle of 45° and ZnSe lens. Spectra were recorded in the range of 600–4000 cm^−1^ at 32 scans with a resolution of 4 cm^−1^.

### 2.9. Contact Angle Measurements

Water contact angles of the fibrous scaffolds were measured with a Ramé-hart goniometer using the sessile drop method. The samples were attached to a glass slide and placed in the sample stage. A droplet of deionized water (10 μL) was automatically dispersed onto the sample surface and its evolution with time was recorded using a CCD video camera attached to the equipment. From the film frames, the water contact angles along time were automatically calculated by the equipment software.

### 2.10. Water Uptake Measurements

Electrospun GelMA scaffolds discs (1 cm in diameter) were cut and weighted (Wo). Then, samples were immersed in PBS at room temperature. After predetermined immersion times, mats were retrieved and weighted (Wt). The water retention (WR) at time t was calculated according to the following equation:

WR(%) = (Wt − Wo)/Wo × 100,(1)

### 2.11. Tensile Testing

Tensile properties of the electrospun scaffolds were tested by using an Instron 4467 universal testing machine (Instron, Norwood, MA, USA). Prior to uniaxial tensile testing, the electrospun fibrous sheets were cut into rectangular shapes (50 mm × 10 mm). Samples were secured between opposing clamps which were approximately 30 mm apart from each other. For tensile testing, samples were stretched until failure at 10 mm/min.

### 2.12. Thermal Analysis

Thermal properties were determined by thermogravimetric analysis (TGA) and differential scanning calorimetry (DSC). TGA were performed on a Shimadzu TGA-50 analyzer from ambient temperature to 300 °C at 10 °C/min under nitrogen atmosphere. DSC thermograms were obtained in a Perkin-Elmer Pyris 1 calorimeter (PerkinElmer Inc., Waltham, MA, USA). Scans were carried out from 25 to 300 °C at a heating rate of 10 °C/min under a nitrogen atmosphere.

### 2.13. X-ray Diffraction

The XRD patterns were obtained using a X-ray diffractometer (PANalytical Model X’pert PRO, Royston, UK). Film samples with dimensions of 4.0 cm × 1.5 cm were cut and fixed in a circular clamp of the instrument. The analysis was carried out directly and the conditions were as follows: (i) voltage and current: 40 kV and 40 mA, respectively; (ii) scan range from 3° to 30°; (iii) step: 0.1° and (iv) speed 1°/min, equipped with a secondary monochromator of graphite beam. The samples were stored at 25 °C and 50% Relative humidity (RH) and analyzed in triplicate.

### 2.14. Roughness Measurements

Roughness was measured by using a laser profilometer (UBM™, ISC-2). A measurement length of 5–7 mm was used with a scanning velocity of 400 points per second. The roughness was calculated using the LMT Surface View UBM™ software.

### 2.15. Cell Viability

For cell culture studies MG-63 osteoblast-like cells (Sigma-Aldrich, Darmstadt, Germany) as an adequate model for bone cells were used [25]. Culture medium Dulbecco’s modified Eagle’s medium (DMEM, Gibco, Darmstadt, Germany) supplemented with 10% (v/v) fetal bovine serum (FBS, Merck, Darmstadt, Germany) and 1% (v/v) penicillin/streptomycin (PS, Merck, Darmstadt, Germany) was chosen. The electrospun GelMA samples were cut into pieces at a diameter of 10 mm and then placed onto the bottom of the culture plates, followed by sterilization under UV light. MG-63 cells were seeded onto the samples in 24-well plate at a density of 1 × 10^4^ cells per well, and conserved into an incubator at 37 °C in a humidified atmosphere of 95% air and 5% CO_2_ for 48 h. After cell culture, the cell viability was determinate by the enzymatic conversion of tetrazolium salt (WST-8 assay, Sigma-Aldrich) to formazan. A volume of 1 mL of a solution of 1% WST-8 assay n cell culture medium was added to each sample, which were incubated for 4 h. The absorbance at 450 nm was measured with a plate reader (typo Phomo, Anthos Mikrosysteme GmbH, Krefeld, Germany). As a blank value, the cell media containing 1% WST-8 without contact to a sample, was used and measured after 4 h of incubation.

## 3. Results

### 3.1. GelMA Nanofibrous Mats

#### 3.1.1. Synthesis of GelMA

GelMA was synthesized according to previously reported methods, in which methacrylate functional groups were grafted onto the gelatin backbone through reactions between methacrylic anhydride and lysine residues [21]. GelMA infrared spectrum showed peaks at 1645, 1526, and 1240 cm^−1^ related to the C=O stretching (amide I), N–H bending (amide II), and C–N stretching plus N–H bending (amide III), respectively. Moreover, a N–H stretching (amide A) could be observed at 3284 cm^−1^. The modification of lysine residues with methacrylate groups was confirmed by a decrease in the lysine signal at 2.9 ppm, and the appearance of the methacrylate group signal at 5.4 ppm and 5.7 ppm and the methyl group signal at 1.8 ppm. A degree of methacrylation of 71% was calculated using Habeeb’s test [24].

#### 3.1.2. Fabrication of Electrospun GelMA Matrices

**Figure 1:**
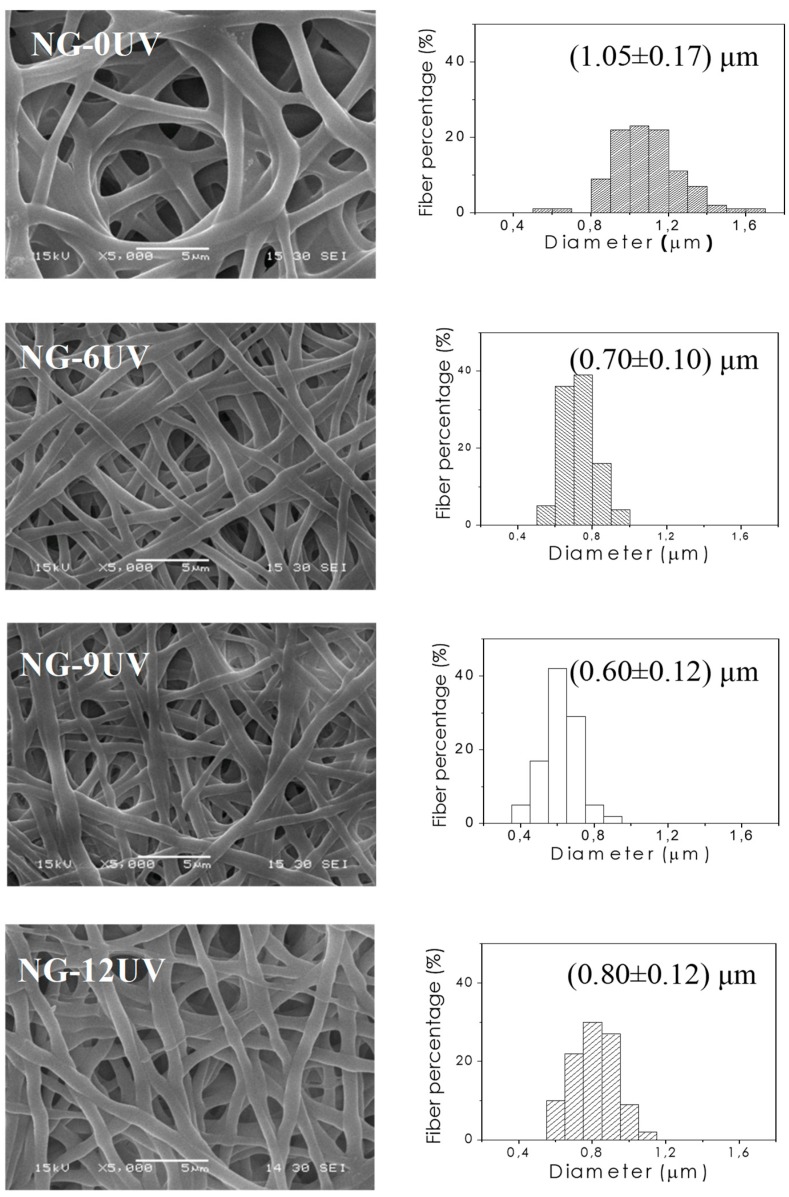
SEM micrographs of samples at different irradiation times: 0 (NG-OUV), 6 (NG-6UV), 9 (NG-9UV) and 12 min (NG-12UV).

Electrospun GelMA mats were prepared by electrospinning technique. Defect-free nanofibrous matrices were obtained as shown in Figure 1 (NG-0UV). Then, electrospun mats were successfully crosslinked by UV irradiation. As it is well-known, photo-chemical crosslinking takes place only in the presence of a photoinitiator, and thus hydrogel formation is triggered by the external source of UV light. Irgacure 2959 is usually chosen as photoinitiator due to its low toxicity at the used concentration as demonstrated in previous studies [23]. To evaluate the UV irradiation process time, GelMA mats were immersed in ethanol and exposed at different irradiation times. Figure 1 shows SEM micrographs of GelMA mats with 0, 6, 9 and 12 min of UV irradiation. In a first step of UV irradiation, fibers increased their diameter as result of their swelling in polar solvents. After UV irradiation, mats still exhibited their fibrous structures and their diameters were reduced with respect to the diameter of NG-0UV. Probably, this decrease in fiber size is related to the crosslinking of GelMA. The increase of diameter in NG-12UV could be ascribed to the increase in temperature produces by UV device.

The mean fiber diameter is summarized in Table 1.

**Figure 2:**
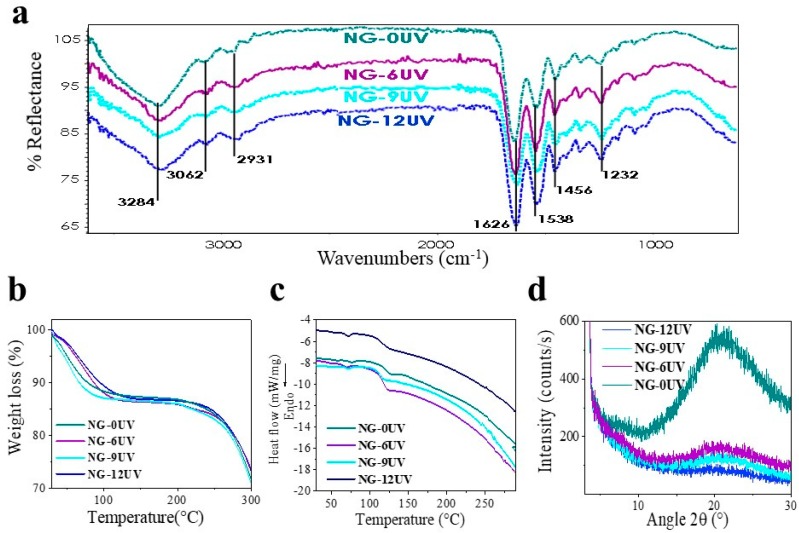
Characterization of samples by (**a**) ATR-FTIR, (**b**) TGA, (**c**) DSC, (**d**) XRD.

The FTIR spectra of GelMA mats showed typical peaks of gelatin (Figure 2a) as described for GelMA. On the other hand, TGA thermograms in Figure 2b show the thermal degradation behavior of GelMA mats. All samples showed a small loss of mass in the range of 50 °C to 120 °C, probably caused by the loss of water molecules. At temperatures above 150 °C, the degradation of biopolymer started for all samples. In addition, their onset points of degradation increased slightly with the time exposed to UV irradiation. Probably, it is due to an increase in the crosslinking degree.

Differential scanning calorimetry (DSC) curves of electrospun GelMA mats are shown in Figure 2c. All samples displayed two endothermic peaks at around 50 and 70 °C. The first peak could be attributed to glass transition of amino acid blocks in the peptide chain relating to the amorphous regions of gelatin. Uncrosslinked gelatin showed a higher Tg than the observed in crosslinked gelatin due to peptide chains, could interact physically by intermolecular bonds. In addition, Tg of GelMA increased with the crosslinking percentage because of the reduced polymeric chain mobility. As suggested in the literature, the endothermic peak at around 70 °C is attributed to denaturation protein [26]. The high Tm of uncrosslinked gelatin could be associated to physical crosslinking of peptide chains. All samples showed endotherm falling at around 97 °C; this is attributed to water loss and degradation of protein.

In order to correlate thermal behavior with structural organization, XRD patterns of samples were obtained (Figure 2d). In spite, gelatin has a crystalline structure originated from α-helix and triple helical; the amorphous structures were observed for all the samples. Probably, the electrospinning processing hinders the re-crystallization of gelatin and the XRD pattern showed an amorphous halo. This peak could be associated with the short range order of protein chains. In addition, the intensity of amorphous peak decreased with the increase in crosslinking degree. Probably, the crosslinking hindered the peptide chains re-ordering.

**Figure 3:**
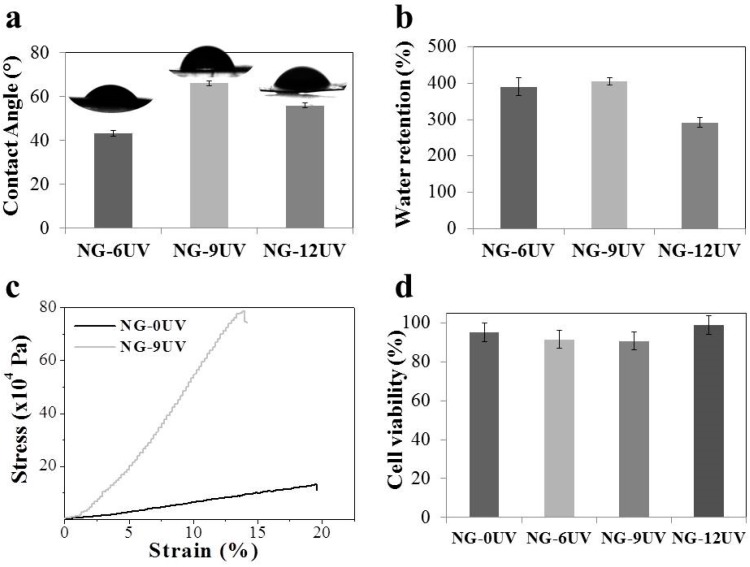
Results of (**a**) CA, (**b**) WR, (**c**) Tensile properties, (**d**) cell viability of GelMA matrices.

The hydrophilicity of the electrospun meshes was evaluated by measuring their water contact angles (Figure 3a, Table 1). The contact angle of uncrosslinked gelatin mat could not be measured because it was absorbed instantaneously when the PBS solution was in contact. However, crosslinked gelatin nanofibers are stable at these conditions. As expected, the CA values of crosslinked gelatin confirm the hydrophilic character of matrices. UV irradiation time could affect not only crosslinking extension, but could also modify the structure, as can be seen in SEM images. Thus, CA values are determined by both chemical and morphological properties.

Although water uptake measurements were carried out for all samples, only crosslinked gelatin electrospun mats were stable in the PBS solution (Figure 3b, Table 1). Swelling percentages of the matrices were around 300%, demonstrating the high hydrophilic character and wettability of matrices. There were no significant differences between water uptake values of NG-6UV and NG-9UV samples. However, NG-12UV showed the lowest swelling degree. This fact can be probably ascribed to the major crosslinking extension of this sample, which clearly affects the water uptake capability.

In order to confirm the crosslinking, the mechanical properties of NG-9UV and NG-0UV nanofiber mats were tested (Figure 3c). A uniaxial tensile testing was performed and the Young’s modulus (YM) was determined for both matrices. YM value of NG-9UV sample was seven times higher than the measured for NG-0UV (0.717 ± 0.001 MPa and 4.89 ± 0.03 MPa, respectively). Thus, the crosslinking methodology used in this work for GelMA curing was successful. Moreover, a relationship between the morphological/chemical properties and UV irradiation time was observed. Thus, a UV irradiation time of 9 min was chosen for further studies, according to the obtained results.

### 3.2. Micropatterned Nanofibrous GelMA Scaffolds

#### 3.2.1. Fabrication of Micropatterned Molds

**Figure 4:**
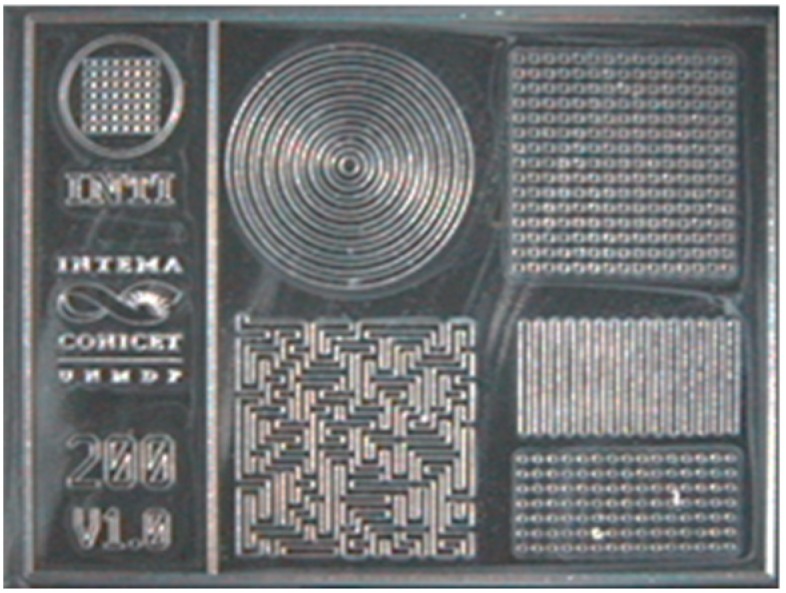
Microscope images of micropatterned mold.

3D structures were created by optical lithography of epoxy-based photoresist on silicon wafers, obtaining 115 µm thick molds. Designs included patterns of 50 µm, 100 µm, 200 µm, and 400 µm. Figure 4 shows a microscope image (BX 51, Olympus) of a chip with 200 µm structures.

#### 3.2.2. Fabrication of Micropatterned Nanofibrous GelMA Scaffolds

**Figure 5:**
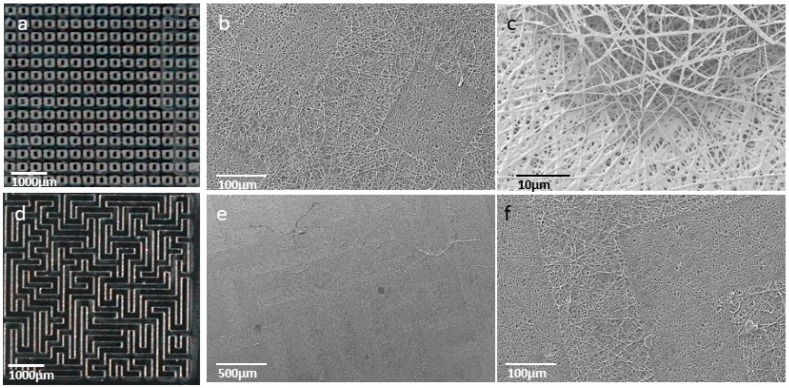
Optic images of 200 µm row and maze patterned molds (**a** and **d**, respectively) and SEM images of 200 µm patterned GelMA electrospun mats (**b** and **c**: row patterns; **e** and **f**: maze patterns).

Once molds were fabricated, micropatterned nanofibrous scaffolds were obtained. The electrospun mats were collected over micropatterned molds and then they were peeled out carefully. GelMA nanofibrous mats were obtained using five different types of surface topographies: concentric-circles, dot, parallel-lines, random-lines (maze), and squares. After crosslinking, samples were examined by SEM. The electrospun mats successfully reproduced the micropatterned design of the molds, keeping the nanofibrous structure when the size of structures are 200 µm or more. Two of the micropatterned designs are shown in Figure 5.

Surface roughness is among the key parameters for biomaterials, which affect the cell attachment and proliferation. Roughness measurements were carried out on the patterned GelMA electrospun mats (Table 2). Fibers produced with 400 μm of thickness (NG-P400) were rougher than the sample produced with 200 μm of thickness (NG-P200). The depth of the patterns on the mats can be estimated from the roughness values recorded at Z-axis. The depth of the step on the mat NG-P400 was around 4 µm, whereas the depth of the step on the NG-P200 was estimated to be 2.5 µm. For all GelMA fiber mats with different topographies, the measured values of roughness showed an increase of surface patterning.

### 3.3. Cell Viability

GelMA electrospun mats were tested in vitro to determine the biocompatible character of the process (Figure 3d). Cell viability percentages of all samples were above 90%. The high number of living cells indicates that the crosslinking process and the modified gelatin were compatible with the used cell-line MG-63. Thus, gelatin based electrospun mat with micro- and nanotopographical features have a great potential for tissue engineering.

## 4. Discussion

The use of gelatin as a biopolymer scaffolding material for tissue engineering applications is directly related to its high biocompatibility, hydrophilicity, and bioactivity associated with specific peptide sequences. Electrospinning is a very attractive, complex, and versatile technique to prepare advanced functional nanofibrous scaffolds for a variety of tissue engineering and cell culture applications. Current efforts are focused on preparing electrospun scaffolds with controlled multilevel hierarchical structures. GelMA electrospun scaffolds have been reported using synthetic polymers and/or hazard, expensive solvents [27,28,29]. However, a pure gelatin scaffold that mimics the important features of natural ECM obtained by electrospinning is still pending. In this work, GelMA nanofibers without defects were obtained using acetic acid, a low toxic potential solvent. Furthermore, in order to overcome the limitations of the low stability of gelatin in aqueous media as well as the possible toxicity of chemical crosslinkers, the as-spun scaffolds were crosslinked by UV irradiation at different exposition times. The same strategy, but while varying the photoinitiator concentration, was reported by Lai et al. for cultivation of limbal epithelial cells [30].

The GelMA matrix was soluble in PBS before UV irradiation (NG-0UV) while crosslinked GelMA nanofibers were stable in PBS (NG-6UV, NG-9UV and NG-12UV). The mechanical properties of UV irradiated mats (NG-9UV) increased 7 times related to mats unexposed to UV (NG-0UV), indicating the efficiency of crosslinking.

In the last few decades, surface engineering technologies have been used as important tools to clarify the effects of the microenvironment on cellular behavior [3,11,12,13,14]. The fabrication of the surface topographies with geometrical micro and nanopatterns, like channels, pillars and pits with controlled dimensions have been possible through the use of various methods as photolithography, electron beam lithography and microfluidics. These geometric and topographical factors can have an influence in cell adhesion, migration, differentiation, and the shape of cells. To modulate cell behavior through surface engineering methods is not only useful to stimuli stem cell differentiation, but also to generate a favorable response of the implant. In order to control substrate topography, the micropatterns molding with different geometries and dimensions, were developed to fabricate scaffolds. Molds were obtained by photolithography and they were used as collector in electrospinning process. Therefore, GelMA electrospun mats were obtained with nano structure and microroughness. The SEM images and roughness analysis showed that micropatterns were successfully copied over GelMA electrospun mats. To the best of our knowledge, this is the first report in literature in which GelMA nanofibrous scaffolds with micropattern topographical features were designed and prepared.

By combining molding and electrospinning processing, it was possible to obtain scaffolds which mimic ECM. Thus, this simple and easy technique can be useful for developing sophisticated and complex materials for tissue engineering and cell culture. In future works, we will study deeply how different patterns influence the behavior of specific cell lines.

## 5. Conclusions

In summary, an inexpensive and rapid method for the fabrication of well-defined micro- and nanotopographic features on uniform bead-free electrospun GelMA fibers were developed in order to design 3D scaffolds that mimic ECM. The synthesis of GelMA and its processing by electrospinning was optimized, so therefore, defect-free GelMA nanofibrous mats were obtained. The results showed that crosslinking took place successfully and it could modulate the final properties. Furthermore, the use of micropatterned molds obtained by photolithography as a collector in the electrospinning process allowed controlling the roughness of mats. Taking into account the cell viability results, the methodology used in this study is a valuable tool to develop patterned GelMA based nanofibrous scaffolds for cell culture and tissue engineering.

## Figures and Tables

**Table 1:** Fiber diameter, contact angle, water uptake (WR), and DSC results, melting temperature (Tm), and the onset of glass transition temperature (Tg), of GelMA mats.

Matrix.	Mean Diameter (µm)	Contact Angle (°)	WR (%)	Tg_onset_ (°C)	Tm_onset_ (°C)
NG-0UV	1.05 ± 0.17	0	n/d	57.6	74.1
NG-6UV	0.70 ± 0.10	43.2	390 ± 21	52.3	66.5
NG-9UV	0.60 ± 0.12	66.1	405 ± 10	52.9	71.2
NG-12UV	0.80 ± 0.12	56.0	290 ± 15	53.9	68.6

**Table 2:** Roughness values ^1^ of patterned GelMA electrospun mats.

Sample	Ra (µm)	Rz (µm)	Rmax (µm)
**NG-P400**	1.5 ± 0.5	4.0 ± 0.2	14 ± 1
**NG-P200**	0.7 ± 0.05	2.5 ± 0.4	6 ± 1

^1^ Ra: arithmetical mean deviation of the assessed profile, Rz: arithmetical mean deviation of the assessed profile, and Rmax: maximum peak-to-valley height.
